# Notes and News

**Published:** 1897-07-31

**Authors:** 


### Notes and News.

(Collected and Communicated.)

Lord Rothschild has been elected president of the Royal Hospital for Diseases of the Chest, City Road.

The ceremony of laying the foundation-stone of the eleventh block of houses in connection with the Royal National Hospital for Consumption, Ventnor, will be performed by H.R.H. Princess Henry of Battenberg on Saturday, July 31st, at four o'clock.

Hospital Saturday was held in Sydney on May 8th, and the amount received up to date, including indoor and outdoor collections, has reached £3,720, being a slight decrease compared with last year. The same large army of ladies paraded and besieged the streets of city and suburbs. The number of small value coins —halfpennies, pennies, and threepenny-pieces—was greatly in excess of former years.

The following sums have been paid into the Prince of Wales's Hospital Fund: A donation of £250 and an annual subscription of £50 from Messrs. Debenham and Freebody, of Wigmore Street; Mr. William McEwan, a contribution of £1,000; In Memoriam, J. M. (in small sums), £50; and H.H. the Raja of Khetri, as a thankoffering on the occasion of the Diamond Jubilee, £100.

The Vestries of Lambeth, Camberwell, and Battersea have received a letter from Messrs. Busk and Mellor, solicitors, stating that one of their clients (who does not wish his name to appear in the matter) is prepared to contribute to the provision of a large general hospital the sum of £10,000 upon condition that the site of the said hospital shall be in either of the parishes of Lambeth, Camberwell, or Battersea, that the contract for the erection of such hospital shall be entered into not later than August, 1899, and that no medical school shall at any time be formed or carried on in connection with such hospital. The letter goes on to state that their client's idea is that the control and administration of such hospital shall be vested in the three parishes referred to, and that the funds shall be invested in the names of (say) nine trustees, three to be chosen by each parish. The letter will be discussed at the next meeting of the Vestries concerned.

Next Sunday, the 7th Sunday after Trinity, is named by the Church Sanitary Association as suitable for stimulating from the pulpit the efforts now being made by sanitary authorities and others in the direction of providing wholesome surroundings for all.

The admission of H.R.H. the Prince of Wales to the Honorary Fellowship of the Royal College of Physicians is perhaps the highest compliment that could be paid by Royalty to the *personnel* of the medical profession. Honours granted to individuals who have made themselves famous for what they have done to promote our knowledge of disease, or to increase our means of curing it, are always welcome, as showing that progress in medical science is appreciated in high quarters and that the Fountain of Honour is not unmindful of the fact that work done in preventing suffering, in lengthening life, and in curing disease is worthy of the highest recognition. The action of the Prince of Wales, however, in personally allying himself with those whose lives are devoted to medical work is a mark of sympathy with the profession at large—with medical men as well as with medical science—which we feel sure will be highly appreciated throughout the length and breadth of the land.

At the last chapter of the Guild of St. Luke a discussion was held on the proposed residential college for those intending to go as medical missionaries in connection with the Church of England. It was proposed to found a college in London at which students studying medicine at the London hospitals might live, a certain number of whom would be supported by the guild or other societies, and when qualified would be expected to go as medical missionaries, but other students who had no such intention would also be received. In view of the proposed reconstruction of the University of London, the scheme is of special interest, as it may contain the germ of a class of residential colleges. The college of St. Luke would be governed by a principal, who would be a medical man, and by a chaplain. Amongst the speakers were Dr. Symes Thompson, Dr. Russell Wells, Mr. Devereux Marshall, the Bishop of Bloemfontein, the Bishop of Lebombo, and the Rev. C. H. Rouse.

A movement is on foot in Sydney to establish a consumptive hospital and consumptive homes as a memento of the Queen's Jubilee. The proposed name is "The Queen Victoria Hospital and Homes." A large number of people attended at a public meeting held in the Town Hall to consider the matter, at which the Mayor of Sydney presided; and the Governor (Lord Hampden) proposed the first resolution, which was to the effect that a hospital and homes be established for consumptives. A large and influential committee has been formed. The Hon. Alice Brand and H. S. Levy are hon. treasurers, and Miss Fairfax and W. Cecil Purser are hon. secretaries. A sum of £3,100 has already been subscribed, towards which John Fairfax and Sons have subscribed £1,000, and Walter Hall, Esq., £600. Up to the present time there has been no special provision made for consumptives in New South Wales, nor, in fact, in any of the Colonies, with the exception of the one magnificent work of Colonel and Mrs. Goodlet, who have provided and cared for some hundreds of phthisical patients at Thirlmere, Picton, W.S.W., for several years past. Thirlmere is in reality a cottage home, and provides accommodation for some 36 patients. Other than this, the only refuge is the Asylums for Infirm and Destitute.

